# A fatal course of hemophagocytic lymphohistiocytosis in a child with homozygous ERCC6L2 defect and heterozygous ADA2 variant: a case report

**DOI:** 10.3389/fimmu.2025.1717567

**Published:** 2026-02-03

**Authors:** Szymon Lulek, Katarzyna Bąbol-Pokora, Monika Radwańska, Daniel Popiel, Magdalena Reich, Wojciech Młynarski, Radosław Chaber

**Affiliations:** 1 Students’ Scientific Association at the Department of Pediatric Hematology and Oncology, Department of Pediatrics, University of Rzeszów, Rzeszów, Poland; 2 Department of Pediatrics, Oncology and Hematology, Medical University of Lodz, Lodz, Poland; 3 Clinic of Pediatric Oncology and Hematology, State Hospital 2, Rzeszow, Poland; 4 Clinic of Intensive Care and Anesthesiology, State Hospital 2, Rzeszow, Poland; 5 Clinic of Pediatrics and Pediatric Gastroenterology, State Hospital 2, Rzeszow, Poland; 6 Department of Pediatrics, Faculty of Medicine, University of Rzeszow, Rzeszow, Poland

**Keywords:** hemophagocytic lymphohistiocytosis (HLH), ERCC6L2, ADA2/DADA2 deficiency, cytopenias, immunodeficiency, genetic predisposition, hyperinflammation

## Abstract

Hemophagocytic lymphohistiocytosis (HLH) is a life-threatening hyperinflammatory syndrome that may arise secondary to genetic or acquired triggers. Although HLH has been reported in patients with adenosine deaminase 2 (ADA2) deficiency, to date it has not been reported in individuals harboring pathogenic variants in *ERCC6L2*, a gene typically linked to inherited bone marrow failure. We report a fatal case of HLH in a 2-year-old girl with persistent fever, cytopenias, hepatosplenomegaly, liver failure, and multiorgan dysfunction. Despite targeted HLH therapy, the disease progressed rapidly. Genetic testing identified a homozygous pathogenic variant in *ERCC6L2* and a heterozygous *ADA2* variant, which we interpret as indicating a susceptibility background to immune dysregulation, with HLH most plausibly occurring as a secondary, trigger-dependent event. No functional validation was performed, and causal inference cannot be made on this basis. To our knowledge, this is the first documented case of HLH in a patient with a homozygous pathogenic *ERCC6L2* variant. The co-occurrence of a heterozygous *ADA2* variant may have modulated the hyperinflammatory response. These observations highlight the importance of genetic evaluation and suggest—while not proving—a broader spectrum of genetic contexts associated with pediatric HLH; confirmation will require functional studies and replication.

## 1 Introduction

Hemophagocytic lymphohistiocytosis (HLH) is a life-threatening hyperinflammatory syndrome characterized by persistent fever, cytopenias, hepatosplenomegaly, coagulopathy, and markedly elevated biomarkers, including ferritin and soluble interleukin-2 receptor (sIL-2R). HLH results from dysregulated immune responses, particularly impaired granule-mediated cytotoxicity of NK and CD8+ T lymphocytes, leading to uncontrolled macrophage activation and excessive cytokine release. The disorder is classified into primary forms—associated with mutations in genes such as *PRF1*, *UNC13D*, *STX11*, and *STXBP2*—and secondary forms triggered by infections, malignancies, or autoimmune conditions (1).

Excision repair cross-complementing 6-like 2 (*ERCC6L2*) has recently been recognized as a critical gene implicated in inherited bone marrow failure (BMF) syndromes. Biallelic germline defects in *ERCC6L2* impair DNA repair and increase oxidative stress. The clinical course is typically characterized by erythroid predominance and progression to acute erythroid leukemia, which carries a poor prognosis (2).

Pathogenic variants *ADA2* have been associated with autoinflammatory disorders, including HLH (3). Unlike ADA1 deficiency (loss of intracellular ADA), ADA2 deficiency results from malfunction of the extracellular, growth factor-like enzyme, which is required for myeloid differentiation and immune regulation. HLH in the context of *ADA2* variants shows variable severity and is often precipitated by infectious triggers (4).

Here, we report a 2-year-old girl with severe HLH in whom we identified a homozygous pathogenic *ERCC6L2* variant together with a heterozygous *ADA2* variant. To our knowledge, this is the first documented case of HLH in a patient with a homozygous pathogenic *ERCC6L2* variant.

## 2 Case report

A 2-year-old girl was admitted with persistent fever lasting more than five days, progressive apathy, poor oral intake, and markedly reduced urine output.

At 7 months of age she experienced severe COVID-19 pneumonia; at 10 months, roseola infantum complicated by leukopenia and thrombocytopenia; and at 20 months, severe varicella with secondary bacterial skin infection. Prior records documented transient, infection-associated abnormalities in peripheral blood counts during earlier illnesses. Between episodes, values returned to age-adjusted reference ranges, and no transfusion dependence was noted. One week before admission, she received the measles–mumps–rubella (MMR) vaccine. Family history was notable for the sudden death of a male relative (the child of a paternal great-uncle) following polio vaccination.

The patient appeared severely ill and somnolent, with periorbital and peripheral edema, jaundice, and tachycardia (150–160 bpm). The abdomen was distended with palpable splenomegaly.

Initial tests revealed profound leukopenia (WBC 2.25 ×10^9^/L), thrombocytopenia (PLT 63 ×10^9^/L), anemia (HGB 91 g/L), and markedly elevated inflammatory markers: CRP >100 mg/l; (ref. <10); procalcitonin 4.78 µg/L, (ref. <0.5); IL-6 2,061.6 ng/L (ref. < 4.4). Metabolic acidosis was evident: HCO_3_^−^ 15.4 mmol/l, (ref. 21,2-27,0); lactate 2.2–2.4 mmol/L, (ref. 0,56–1,33). Liver dysfunction was severe, with elevated aminotransferases: ALT 171 U/L, (ref. 13 – 45); AST 535 U/L, (ref. 28 – 57), hyperbilirubinemia: total bilirubin 103 μmol/L (ref. 5.1–20.5), and hypoalbuminemia: 23.8 g/L, (ref. 34 – 42). Coagulopathy was indicated by prolonged INR (1.62), low fibrinogen: 1.07 g/l, (ref. 2,0 - 4,0); and markedly elevated D-dimers: 54.2 mg/L, (ref. 0-0.5). Ferritin exceeded 4,000 μg/L, (ref. 10-290).

Abdominal ultrasound demonstrated splenomegaly, gallbladder wall thickening, increased renal cortical echogenicity, and ascites. By day 11 of hospitalization, hepatomegaly was also observed. Chest CT revealed bilateral perihilar infiltrates and echocardiography identified a moderate pericardial effusion.

Blood culture grew *Staphylococcus hominis* (methicillin-resistant coagulase-negative staphylococcus, MRCoNS). Serology was negative for *Mycoplasma*, EBV, HAV, HBV, HIV, Rotavirus, Adenovirus, and *Campylobacter* but positive for CMV IgG and Parvovirus B19 IgG.

Bone marrow aspiration revealed hypocellular marrow with absent megakaryocytes. The erythroid lineage (6%) showed normoblastic maturation; granulocytes (55%) displayed dysplastic features; lymphocytes accounted for 37%. Overall, findings were consistent with an aplastic marrow pattern. No hemophagocytosis was observed.

Cerebrospinal fluid was clear, straw-colored, with a nucleated cell count of 5 cells/µL, elevated protein (1.314 g/L; ref. 0,15–0,45), and normal glucose and chloride levels. Pandy’s test was positive, the Nonne–Apelt test equivocal, and the Weichbrodt test negative. Cytology showed exclusively mononuclear cells, consistent with blood–brain barrier dysfunction, with no cytologic evidence of purulent meningitis.

Clinical and laboratory findings met the HLH-2004 diagnostic criteria (5)— fever >38.5°C, cytopenias, hypertriglyceridemia, splenomegaly, and hyperferritinemia (≥500 µg/L)— establishing the diagnosis of HLH. Germline testing identified a homozygous pathogenic *ERCC6L2* variant (NM_001010895.2:c.1930C>T; p.(Arg644*)), introducing a premature termination codon at position 644, together with a heterozygous *ADA2* missense variant (NM_001282225.2:c.1078A>G; p.(Thr360Ala)) resulting in a threonine-to-alanine substitution at position 360. No functional assays (e.g., ERCC6L2 transcript/protein quantification, DNA-damage–response readouts or ADA2 enzymatic activity) were performed.

**Figure 1:**
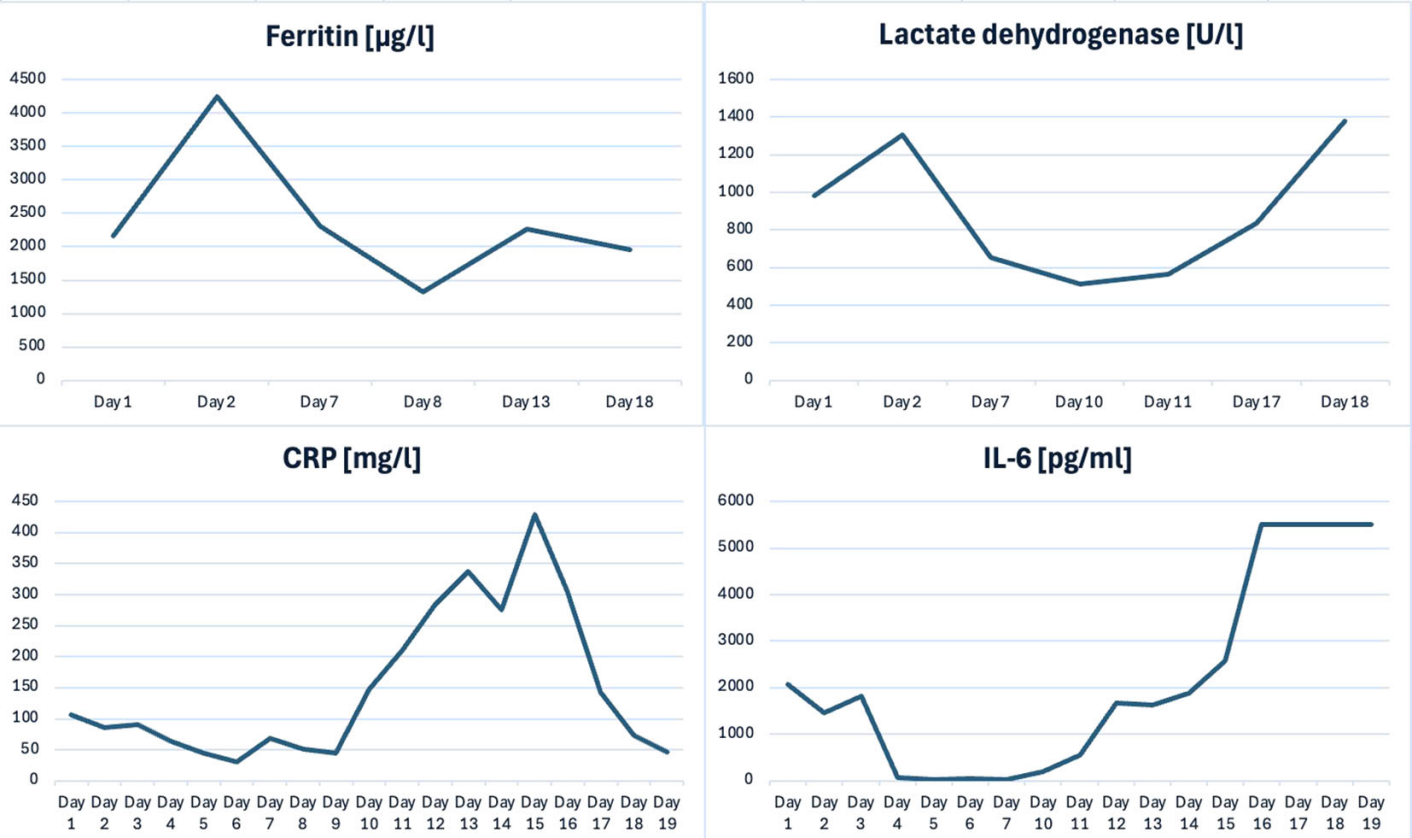
Trends of selected inflammatory and organ function markers.

Despite intensive therapy—including broad-spectrum antibiotics (cefotaxime, vancomycin), antifungals (fluconazole), diuretics, intravenous fluids, albumin infusion, and electrolyte correction—the patient’s condition remained critical. The HLH-2004 regimen was initiated with dexamethasone at 10 mg/m²/day; cyclosporine A at 6 mg/kg/day in two divided doses; and intravenous etoposide (first dose 75 mg/m²/day due to hepatic/renal dysfunction, followed by 150 mg/m²/day). In addition, two doses of tocilizumab (12 mg/kg iv each) were administered as immunomodulatory therapy. Nevertheless, extensive inflammatory changes developed in the lungs. On hospital day 8, acyclovir was introduced for suspected varicella/herpetic reactivation, and antibiotics were escalated to meropenem and amikacin with the addition of amphotericin B. Despite these measures, the child developed progressive multiorgan failure, accompanied by persistently elevated inflammatory markers (**Table 1**, **Figure 1**). On hospital day 19, she suffered cardiac arrest and died.

**Table 1:** Serial laboratory parameters during hospitalization.

laboratory tests	Day one	3rd day	6rd day	9rd day	12rd day	18rd day
White blood cell [K/μl]	2,95	7,16	9,54	1,49	0,32	0.24
Hematocrit [%]	29,60	36,20	31,70	31,70	27,30	30,90
Hemoglobin [g/dl]	10,40	12,50	11,40	10,60	8,90	9,10
Platet Count [K/μl]	22	23	38	75	235	148
Ferritin [μg/l]	2167,70	-	2301,10	-	2260,80	1959,30
Lactate dehydrogenase [U/l]	981,00	–	655,00	514,00	-	1378,00
Fibrinogen [g/l]	2,36	0,87	2,09	-	7,57	0,98
Triglycerides [mmol/l]	252	-	–	-	504	766
Total bilirubin[mg/dl]	5,70	6,30	8,10	2,00	1,10	-
ALT [U/L]	131,00	111,00	90,00	52,00	32,00	-
AST [U/L]	259,00	199,00	78,00	42,00	37,00	-
aPTT [s]	34,60	30,70	25,90	-	30,00	24,30
IL-6 [pg/ml]	2061,60	25,60	30,90	84,50	1673,10	>5500
CRP [mg/l]	106,00	52,80	68,70	147,20	337,60	46,10
Procalcitonin (PCT) [ng/ml]	1,72	10,13	1,28	0,33	2,64	35,79

## 3 Discussion

This case illustrates the extreme severity and diagnostic complexity of HLH in the setting of coexisting genetic abnormalities. To our knowledge, this is the first report of HLH in a patient with a homozygous pathogenic *ERCC6L2* variant, accompanied by a heterozygous *ADA2* variant.

HLH may arise sporadically or in association with genetic disorders of immune regulation (6). Familial HLH results from biallelic defects in genes involved in NK- and T-cell cytotoxicity (e.g., *PRF1*, *UNC13D*), whereas secondary HLH typically lacks an identified monogenic defect and is most often triggered by infection or malignancy. However, mounting evidence indicates that some patients categorized as “secondary” HLH carry heterozygous variants in HLH-related genes or other germline immune defects (7). Notably, HLH has rarely been reported in inherited BMF syndromes, although a recent case described HLH as the initial presentation of telomerase-related BMF (8).

Deficiency of DADA2, an autosomal-recessive disorder caused by biallelic loss-of-function variants in *ADA2*, has been identified as one HLH-associated autoinflammatory condition (9). Its clinical spectrum ranges from systemic inflammation to immunodeficiency and cytopenias (10). Hematologic manifestations occur in approximately 20–25% of patients, whereas HLH is rare but severe, reported in <1% of cases (11). Additionally, ADA2 deficiency skews monocytes toward a pro-inflammatory M1 phenotype and disrupts NK/T-cell regulatory interactions, creating a cytokine-rich milieu characterized by elevated TNF-α, IL-6, and type I interferons (12, 13). Moreover, ADA2-deficient cytotoxic lymphocytes exhibit impaired granzyme release, which may predispose to HLH in the context of viral triggers (14).

Classic DADA2 requires biallelic variants, and heterozygous carriers are usually asymptomatic. Exceptions have been reported: Wouters et al. (2024) demonstrated that certain heterozygous variants exert dominant-negative effects on ADA2 activity, dimerization, or secretion (15). Several reports describe symptomatic heterozygous carriers with presentations ranging from immunodeficiency to inflammatory and vascular phenotypes (16–19). Pulvirenti et al. (2023) reported a heterozygous carrier who developed fatal multiorgan failure, supporting the hypothesis that single-allele pathogenic variants may contribute to severe inflammation (10). In our patient, the heterozygous *ADA2* variant alone does not fulfil criteria for DADA2 but may have facilitated immune dysregulation. Partial ADA2 impairment could have lowered the threshold for hyperinflammation, acting synergistically with *ERCC6L2*-related marrow stress as a “second hit.”

*ERCC6L2* encodes a helicase central to transcription-coupled nucleotide-excision repair and the genomic integrity of hematopoietic stem cells (20). Biallelic germline variants cause an inherited BMF syndrome that often progresses to MDS/AML. Moreover, approximately 90% of affected patients acquire somatic *TP53* variations during the BMF/MDS phase, driving rapid evolution to high-risk AML with a median survival of 3 months after transformation (2, 21, 22). HLH has not previously been described in ERCC6L2-related disease, but sustained marrow stress and apoptotic turnover could leave cytotoxic effectors quantitatively and functionally compromised, favoring dysregulated macrophage activation. Consistent with this framework, preclinical ERCC6L2-deficiency models show upregulated TP53 signaling and remodeling of the marrow microenvironment (23). Although these changes do not implicate ERCC6L2 in the perforin–granule pathway that defines classic familial HLH, they may lower the threshold for infection- or vaccine-driven hyperinflammation. Taken together, and in the absence of functional validation or confirmation of *ERCC6L2* loss of function in other patients with HLH, it is not possible to establish a direct link between homozygous *ERCC6L2* variants and the development of HLH. It is plausible that this episode represents vaccine-triggered HLH occurring in the setting of a homozygous pathogenic *ERCC6L2* variant, with a heterozygous *ADA2* variant acting as a potential modifier.

Notably, this case highlights the need for careful pre-vaccination assessment in children with unexplained cytopenias and recurrent infections suggestive of immune dysfunction. In such patients, live-attenuated vaccines can trigger uncontrolled immune activation, warranting genetic evaluation and close clinical monitoring. This observation also supports considering *ERCC6L2* testing in HLH when “classic” pathogenic variants are not identified.

## 4 Conclusions

This fatal case illustrates the genetic complexity and likely multifactorial pathogenesis of HLH. Our literature review identified no previous reports of HLH in ERCC6L2-related bone-marrow failure indicating that this association is exceedingly rare. Although a direct causal relationship between homozygous *ERCC6L2* variants and HLH cannot be established from a single observation, the coexistence of homozygous ERCC6L2 deficiency and a heterozygous *ADA2* variant—together with a recent exposure to a live-attenuated vaccine—may have acted as disease modifiers that lowered the threshold for hyperinflammation. These considerations support including *ERCC6L2* in the genetic work-up of HLH when “classic” predisposing variants are not identified. Further research, including functional studies and replication in independent cases, is needed to determine whether this represents a rare coincidence or whether similar oligogenic gene–environment interactions underlie a subset of currently “idiopathic” HLH.

## Data Availability

The original contributions presented in the study are included in the article/supplementary material. Further inquiries can be directed to the corresponding author.
